# Fibroblast Growth Factor Receptor 2 (FGFR2), a New Gene Involved in the Genesis of Autism Spectrum Disorder

**DOI:** 10.1007/s12017-023-08759-w

**Published:** 2023-09-21

**Authors:** Antonio Gennaro Nicotera, Greta Amore, Maria Concetta Saia, Mirella Vinci, Antonino Musumeci, Valeria Chiavetta, Concetta Federico, Giulia Spoto, Salvatore Saccone, Gabriella Di Rosa, Francesco Calì

**Affiliations:** 1 Department of Human Pathology of the Adult and Developmental Age, “Gaetano Barresi”, University of Messina, Via Consolare Valeria 1, 98125 Messina, Italy; 2 Oasi Research Institute—IRCCS, Via Conte Ruggero 73, 94018 Troina, Italy; 3 Department Biological, Geological and Environmental Sciences, University of Catania, Via Androne 81, 95124 Catania, Italy; 4 Department of Biomedical Sciences, Dental Sciences and Morpho-Functional Imaging, University of Messina, Via Consolare Valeria 1, 98125 Messina, Italy

**Keywords:** Autism, *FGFR2* gene, Intellectual disability, Human chromosome 10, Sanger sequencing, Missense mutation

## Abstract

Autism spectrum disorder (ASD) is a long-known complex neurodevelopmental disorder, and over the past decades, with the enhancement of the research genomic techniques, has been the object of intensive research activity, and many genes involved in the development and functioning of the central nervous system have been related to ASD genesis. Herein, we report a patient with severe ASD carrying a G > A de novo variant in the *FGFR2* gene, determining a missense mutation. *FGFR2* encodes for the ubiquitous fibroblast growth factor receptor (FGFR) type 2, a tyrosine kinase receptor implicated in several biological processes. The mutated version of this protein is known to be responsible for several variable overlapping syndromes. Even if there still is only sparse and anecdotal data, recent research highlighted a potential role of *FGFR2* on neurodevelopment. Our findings provide new insights into the potential causative role of *FGFR2* gene in complex neurodevelopmental disorders.

## Introduction

Autism spectrum disorder (ASD) is a complex neurodevelopmental disorder defined by deficits in social communication and restricted repetitive sensory–motor behaviours. Over the past decades, researchers mainly aimed to unravel the causes underpinning ASD clinical and etiological heterogeneity, and to discover specific biomarkers and endophenotypes (Shen et al., 2020). Although inconclusive, ongoing research has shed light on genetic factors potentially implicated in ASD susceptibility and severity (Hodges et al., 2020). The breakthrough of genome-wide association studies and whole exome sequencing techniques have helped broaden our understanding of ASD-related genes and to find common genetic risk variants (Grove et al., 2019). Several candidate genes seem to play a role in brain development, neurotransmitter function, or neuronal excitability further to the formation and maintenance of neuronal synapses (*i.e. RELN*, *MeCP2*, *NLGN3*, *NLGN4*, *TSC2*, *CDKL5*, *NRXN1*, *VAMP2*) (Hodges et al., 2020; Spoto et al., 2022). Herein, we describe a 12-year-old Sicilian male patient with severe ASD, associated with Attention Deficit/Hyperactivity Disorder (ADHD), carrying a de novo variant in the *fibroblast growth factor receptor 2* (*FGFR2*) gene.

## Materials and Methods

### NGS Technology

Exome analysis was performed as described (Calì et al., 2017). The identified variants were filtered according to (i) recessive/de novo/X-linked pattern of inheritance and (ii) allele frequencies using the following genomic datasets: *1000 Genomes*, *ESP6500*, *ExAC*, and *gnomAD*. In silico analyses were performed with Varsome (ACMG criteria) (Kopanos et al., 2019).

### Sanger Sequencing

Sanger sequencing was performed using the BigDye Terminator v1.1 Cycle Sequencing Kit (Thermo Fisher Scientific) by standard methods.

### Array-CGH

A single-array comparative genomic hybridization (aCGH) experiment on the proband and her parents was performed using the Human Genome CGH Microarray 60 K (Agilent Technologies).

## Results

### Clinical Presentation

We report the case of a 12-year-old boy born to non-consanguineous parents after an uneventful full-term pregnancy. The proband showed delayed psychomotor development (walking at 19 months) and language development restricted to vocalizations, associated with social and behavioural abnormalities (tendency to isolate from peers, stereotypies, the propensity to seek visual stimuli, and toe walking). At 4 years old, according to the Diagnostic and Statistical Manual of Mental Disorders, 5th Edition (DSM-V) criteria, the patient was also diagnosed with autism spectrum disorder. The clinical examination highlighted the presence of plagiocephaly, macrocephaly, macrosomia, arched palate, syndactyly of the 2nd and 3rd toe fingers bilaterally, flat feet, slightly generalized hypotonia, and ligament laxity. Subsequently, a co-diagnosis of severe ADHD was made, and treatment with periciazine was started. At the age of 7 years, despite the pharmacological and habilitative treatments (the child attended Applied Behavioural Analysis and speech therapy and used Picture Exchange Communication System), the patient’s clinical picture gradually worsened (impulsivity, multifarious stereotypies and inadequate participation during treatments). Brain MRI and EEG were both unremarkable. Switching from periciazine to risperidone slightly reduced ADHD and ASD symptoms; moreover, gradually, the child increased in weight and presented several metabolic complications (increased glycated haemoglobin levels and hyperinsulinism). For these reasons, at the age of 10 years, a switch from risperidone to aripiprazole was made, presenting an improvement in behavioural abnormalities, social-communicative skills, and a normalization of the previously reported metabolic disturbances.

### Genetic Analysis

**Fig. 1:**
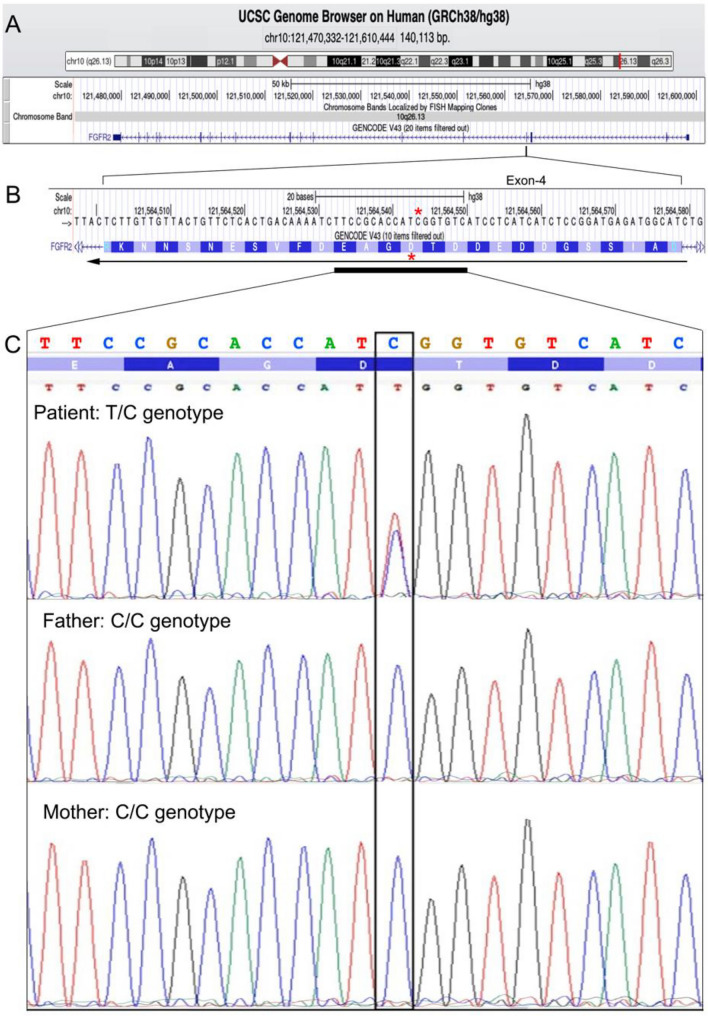
Heterozygous variant identified in the present ASD patient. **A** genomic organization of the *FGFR2* gene localized in the chromosomal band 10q26.13. Gene is transcribed from the telomere to the centromere side and consists of 18 exons. Exon 4 contains the detected C > T variation (G > A in the RefSeq NM_000141.5). **B** Nucleotide and aminoacidic sequences of the *FGFR2* gene exon-4. The red asterisk indicates the nucleotide and the corresponding aminoacidic variant. The horizontal black line indicates the direction of transcription/translation. Data and images from the UCSC Genome Browser (http://genome.ucsc.edu, accessed on 15 June 2023). **C** Sanger sequencing electropherogram of the 21 nucleotides surrounding the C > T variant, showing the heterozygous missense mutation detected in the ASD patient, respect to his parents. A black box highlights the mutated nucleotide

Results from a single aCGH using the Human Genome CGH Microarray 60 K were not informative (data not shown). Trio exome sequencing analysis (WES) was carried out, highlighting a heterozygous variant c.412G > A (p.Asp138Asn) in the *FGFR2* gene [REFSEQ: NM_000141.5]. Using a conventional Sanger sequencing, we confirmed that the variant occurred de novo (Fig. 1c)**.** This SNP was associated in the literature with “Cleft lip and palate” (Riley et al., 2007) and is classified as “likely pathogenic” according to the “American College of Medical Genetics” criteria (Kopanos et al, 2019). In silico analysis was performed with VARSOME. In the absence of any significant variants affecting other genes, we considered the c.412G > A (p.Asp138Asn) in the *FGFR2* gene causative of our patient’s clinical picture.

## Discussion

The ASD patient described here showed a mutation in the *FGFR2* gene, located in the chromosomal band 10q26 (Fig. 1a) and encoding the fibroblast growth factor receptor type 2. It belongs to the family of tyrosine kinase receptors (including FGFR1, FGFR3, and FGFR4) that regulate several biological processes, including bone development, differentiation of mesenchymal and neuroectodermal cells, intracellular survival and proliferative cellular mechanisms (Azoury et al., 2017; Goyal et al., 2021). *FGFR2* has been associated with several heterogeneous autosomal-dominant syndromes, among which the Pfeiffer (#MIM 101600), Crouzon (#MIM 123500), Apert (#MIM 101200) syndromes, variably presenting overlapping features affecting the skin and the skeletal and neurological systems, and often associated with a high risk of cancer. Recently, *FGFR2* has been hypothesized to be implicated in neurodevelopmental disorders as well (Coci et al., 2017; Gracia-Darder et al., 2023; Szczurkowska et al., 2018; Tammimies et al., 2015).

**Fig. 2:**
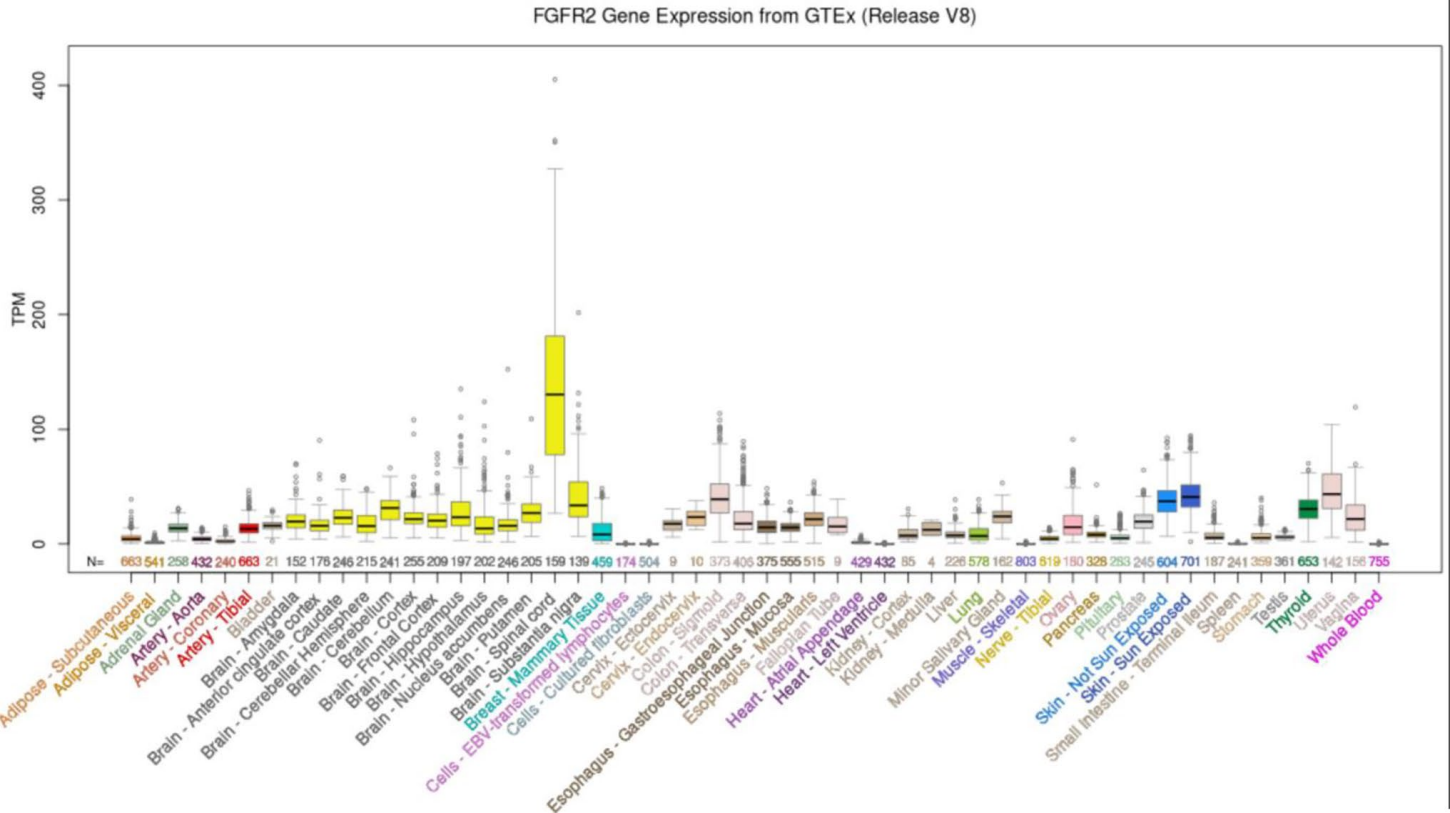
Expression level of the *FGFR2* gene in human healthy tissues. The expression level was obtained in 54 human tissues from GTEx RNA-seq of 17,382 samples from 948 donors (V8, Aug 2019). TPM: Transcripts per Million. Data and image from the UCSC Genome Browser (http://genome.ucsc.edu, Accessed 05 May 2023)

Autosomal-dominant *FGFR2* mutations might result in constitutive activation of the receptor, which, in turn, may determine an early differentiation of the osteoprogenitors, hence, a premature suture fusion (Azoury et al., 2017). Therefore, it is not surprising that most *FGFR2* pathogenic variants (mainly missense ones) lead to syndromic craniosynostosis (i.e., Crouzon, Apert, Pfeiffer, Beare-Stevenson cutis gyrate, Jackson-Weiss, and Seathre-Chotzen-like syndromes) with a multisystemic involvement (Azoury et al., 2017). However, *FGFR2* is nearly ubiquitously expressed, particularly in the brain, respiratory system, male and female tissues, and skin (Fig. 2); thus, mutations in this gene have been associated with other clinical phenotypes, such as Hypospadias and Lacrimo-auriculo-dento-digital syndrome (Azoury et al., 2017; Stenson et al., 2020).

Although the evidence available today is still sparse, recent data unravelled a potential role of *FGFR2* on neurodevelopment, such as the reported presumed loss-of-function mutation (c.A1295G) of *FGFR2* in an autistic child, without features of craniosynostosis (Tammimies et al., 2015**)**. Accordingly, Gracia-Darder et al. (2023) reported a heterozygous variant (p.Cys382Arg) in *FGFR2*, suggesting a possible causative role in the aetiology of their patient’s autism. In addition, Coci et al. (2017) reported three siblings affected by two distinct forms of severe neuropsychological impairments inclusive of autistic features, who turned out to carry two different unbalanced translocations between chromosomes 10 and 22, deriving from a maternal balanced translocation, two chromosomes harbour important genes implicated in central nervous system development and body growth, including the *FGFR2* gene. These data are summarized in Table 1.

**Table 1:** Summary of the clinical features of the patient here described, and comparison with cases from the literature presenting a potential involvement in Autism Spectrum Disorder of the *FGFR2* gene

Feature	Present patient	Tammimies et al. (2015)	Gracia-Darder et al. (2023)	Coci et al. ( 2017) (Patient 1)	Coci et al. (2017) (Patient 2)	Coci et al. (2017) (Patient 3)
Age (years)	12	–	2	13	11	9
Sex	Male	Male	Male	Female	Female	Male
Genetic alteration	SNP	SNP	SNP	Unbalanced translocation: der(10)t(10;22)(q26.13;q13.32)	Unbalanced translocation: der(22)t(10;22)(q26.13;q13.32)	Unbalanced translocation: der(10)t(10;22)(q26.13;q13.32)
Nucleotide change	c.412G > A	c.1295A > G	c.1147 T > C	–	–	–
Effect on protein	p.Asp138Asn	p.Asp432Gly, near splice site	p.Cys382Arg	–	–	–
Inheritance	De novo	Paternal	De novo	Maternal balanced translocation t(10;22)(q26.13;q13.32)	Maternal balanced translocation t(10;22)(q26.13;q13.32)	Maternal balanced translocation t(10;22)(q26.13;q13.32)
Main clinical picture	Severe ASD	ASD	RAVEN	Complex phenotype affecting neurological and skeletal systems, ASD	Intellectual disability	Complex phenotype affecting neurological and skeletal systems
Other associated clinical features	ADHD	Left temporal lobe septated intraparenchymal cyst	Autistic traits	Epilepsy	Autistic traits	Autistic traits, epilepsy
Dysmorphic features	Plagiocephaly, macrocephaly, macrosomia, arched palate, syndactyly of the 2^nd^ and 3^rd^ toe fingers bilaterally, flat feet, generalized hypotonia, and ligament laxity	Dolichocephaly, deep-set eyes	Hair heterochromia	Microcephaly, broad and prominent forehead, flat midface, narrow tip nose, hypotelorism, and strabismus convergent	Macrocephaly	Dolichocephaly, narrow tip nose, hypotelorism, and strabismus convergent)

*ASD* autism spectrum disorder, *ADHD* attention deficit hyperactivity disorder, *FGFR2* fibroblast growth factor receptor 2, *RAVEN* rounded and velvety epidermal nevus

Szczurkowska et al. (2018) have already proposed such a role for *FGFR2* in neurodevelopmental disorders. They demonstrated that the mutual regulation of FGFR2 and the cell adhesion molecule NEGR1 are apparently implicated in the genesis of impaired core behaviours related to ASD in mice: the downregulation of either one of these two genes leads to a defective NEGR1-FGFR2 complex (which in its turn would converge on the Extracellular signal-regulated kinases/Akt downstream signalling pathway), eventually affecting neuronal migration and the spine density during mouse cortical development, being, therefore, responsible for the autistic symptoms. Earlier, Vaccarino et al. (2009), considering that the FGF family is involved in cortical size and connectivity regulation, mini-column pathology and excessive network excitability, hypothesized that mutations in these genes may lead to autistic sensory hyper-reactivity.

Finally, it is worth recalling our patient’s clinical features, partially overlapping with those present in specific *FGFR2*-related syndromes, such as high-arched palate (as in Pfeiffer syndrome), syndactyly of the 2nd and 3rd toes (Jackson-Weiss syndrome #MIM 123150 and Sathre-Chotzen syndrome, #MIM 101400), plagiocephaly (Sathre-Chotzen syndrome), developmental delay, and intellectual disability (Beare-Stevenson cutis gyrate syndrome, #MIM 123790, Crouzon syndrome, and Pfeiffer syndrome).

All these data not only corroborate the already proposed pathogenicity of *FGFR2* in neurodevelopmental disorders, including autism and ADHD but also specifically support its etiological role in our patient’s case. Given the recent breakthrough of NGS techniques, such as WES, we reckon that the analysis of *FGFR2* should be included, and its variants should be taken in mind in patients with neurodevelopmental disorder, especially in association with craniosynostosis and body overgrowth. Further evidence is needed to reveal the specific role of certain FGFR2 variants in determining distinct phenotypes to finally delineate a more precise genotype–phenotype correlation within this rare and intriguing group of disorders.

## Data Availability

Not applicable.

## References

[CR1] Azoury SC, Reddy S, Shukla V, Deng CX (2017). Fibroblast growth factor receptor 2 (FGFR2) mutation related syndromic craniosynostosis. International Journal of Biological Sciences.

[CR2] Calì F, Chiavetta V, Ruggeri G, Piccione M, Selicorni A, Palazzo D, Bonsignore M, Cereda A, Elia M, Failla P, Figura MG, Fiumara A, Maitz S, Luana Mandarà GM, Mattina T, Ragalmuto A, Romano C, Ruggieri M, Salluzzo R, Saporoso A, Schepis C, Sorge G, Spanò M, Tortorella G, Romano V (2017). Mutation spectrum of NF1 gene in Italian patients with neurofibromatosis type 1 using IonTorrent PGM™ platform. European Journal of Medical Genetics.

[CR3] Coci EG, Auhuber A, Langenbach A, Mrasek K, Riedel J, Leenen A, Lücke T, Liehr T (2017). Novel unbalanced translocations affecting the long arms of chromosomes 10 and 22 cause complex syndromes with very severe neurodevelopmental delay, speech impairment, autistic behavior, and epilepsy. Cytogenetic and Genome Research.

[CR4] Goyal L, Kongpetch S, Crolley VE, Bridgewater J (2021). Targeting FGFR inhibition in cholangiocarcinoma. Cancer Treatment Reviews.

[CR5] Gracia-Darder I, Llull Ramos A, Giacaman A, Gómez Bellvert C, Obrador-Hevia A, Jubert Esteve E, Martín-Santiago A (2023). Report of a case of RAVEN, hair heterochromia and autism in the setting of FGFR2 mutation. Pediatric Dermatology.

[CR6] Grove, J., Ripke, S., Als, T. D., Mattheisen, M., Walters, R. K., Won, H., ... & Børglum, A. D. (2019). Identification of common genetic risk variants for autism spectrum disorder. *Nature Genetics**51*, 431-444. Doi: 10.1038/s41588-019-0344-8 10.1038/s41588-019-0344-8 PMC6454898 30804558

[CR7] Hodges H, Fealko C, Soares N (2020). Autism spectrum disorder: Definition, epidemiology, causes, and clinical evaluation. Translational Pediatrics.

[CR8] Kopanos C, Tsiolkas V, Kouris A, Chapple CE, Aguilera MA, Meyer R, Massouras A (2019). VarSome: The human genomic variant search engine. Bioinformatics.

[CR9] Riley BM, Mansilla MA, Ma J, Daack-Hirsch S, Maher BS, Raffensperger LM, Russo ET, Vieira AR, Dodé C, Mohammadi M, Marazita ML (2007). Impaired FGF signaling contributes to cleft lipand palate. Proceedings of the National Academy of Sciences USA.

[CR10] Shen L, Liu X, Zhang H, Lin J, Feng C, Iqbal J (2020). Biomarkers in autism spectrum disorders: Current progress. Clinica Chimica Acta.

[CR11] Spoto G, Valentini G, Saia MC, Butera A, Amore G, Salpietro V, Nicotera AG, Di Rosa G (2022). Synaptopathies in developmental and epileptic encephalopathies: A focus on pre-synaptic dysfunction. Frontiers in Neurology.

[CR12] Stenson PD, Mort M, Ball EV, Chapman M, Evans K, Azevedo L, Hayden M, Heywood S, Millar DS, Phillips AD, Cooper DN (2020). The Human gene mutation database (HGMD®): Optimizing its use in a clinical diagnostic or research setting. Human Genetics.

[CR13] Szczurkowska J, Pischedda F, Pinto B, Manago F, Haas CA, Summa M, Bertorelli R, Papaleo F, Schäfer MK, Piccoli G, Cancedda L (2018). NEGR1 and FGFR2 cooperatively regulate cortical development and core behaviours related to autism disorders in mice. Brain.

[CR14] Tammimies K, Marshall CR, Walker S, Kaur G, Thiruvahindrapuram B (2015). Molecular diagnostic yield of chromosomal microarray analysis and whole-exome sequencing in children with autism spectrum disorder. JAMA.

[CR15] Vaccarino FM, Grigorenko EL, Smith KM, Stevens H (2009). Regulation of cerebral cortical size and neuron number by fibroblast growth factors: Implications for autism. Journal of Autism and Developmental Disorders.

